# A systematic review of homomorphic encryption and its contributions in healthcare industry

**DOI:** 10.1007/s40747-022-00756-z

**Published:** 2022-05-03

**Authors:** Kundan Munjal, Rekha Bhatia

**Affiliations:** 1 Department of Computer Science and Engineering, Punjabi University, Patiala, 147002 India; 2 AIT CSE, Chandigarh University, Gharuan, Mohali 140413 India; 3 Department of Computer Science, Punjabi University, Patiala, 147002 India

**Keywords:** Homomorphic Encryption, Fully Homomorphic Encryption, Health Informatics, Cryptonets, Secure Learning

## Abstract

Cloud computing and cloud storage have contributed to a big shift in data processing and its use. Availability and accessibility of resources with the reduction of substantial work is one of the main reasons for the cloud revolution. With this cloud computing revolution, outsourcing applications are in great demand. The client uses the service by uploading their data to the cloud and finally gets the result by processing it. It benefits users greatly, but it also exposes sensitive data to third-party service providers. In the healthcare industry, patient health records are digital records of a patient’s medical history kept by hospitals or health care providers. Patient health records are stored in data centers for storage and processing. Before doing computations on data, traditional encryption techniques decrypt the data in their original form. As a result, sensitive medical information is lost. Homomorphic encryption can protect sensitive information by allowing data to be processed in an encrypted form such that only encrypted data is accessible to service providers. In this paper, an attempt is made to present a systematic review of homomorphic cryptosystems with its categorization and evolution over time. In addition, this paper also includes a review of homomorphic cryptosystem contributions in healthcare.

## Introduction

**Fig. 1:**
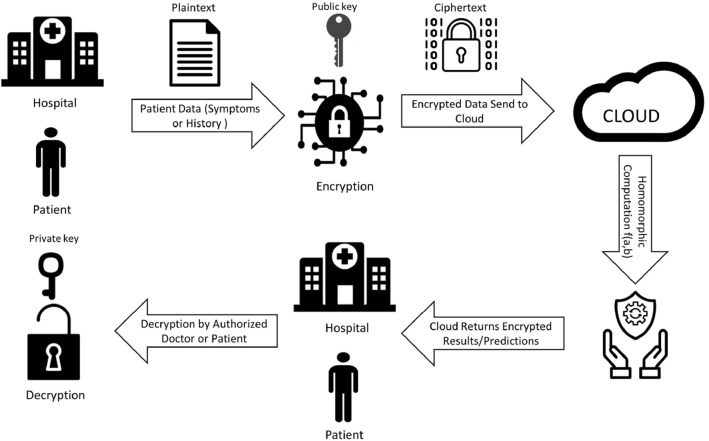
Homomorphic encryption in healthcare

Encryption approaches usually do not allow processing on encrypted data which implies every time data are processed in its original form. On the other hand, homomorphic encryption allows computation on encrypted data and provides encrypted results to the user. Homomorphic encryption not only allows the processing of encrypted data but also preserves privacy in the process. Classic public-key encryption consists of three algorithms: *Key generation*, *Encryption*, and *Decryption*; however, in addition to these three conventional approaches, a homomorphic encryption property is required. *Homomorphic property* $\varepsilon$ *has an efficient algorithm* $Eval_{\varepsilon }$ *with input as* $p_k$ *encryption key,* *C* *is circuit generated from set* $C_\varepsilon$ *and ciphertexts* $C_p = (c_{p1},c_{p2},....c_{pt})$ *encrypted under public key* $p_k$ *then* $Eval_{\varepsilon }(p_k,C,C_p)$ *encrypts* $C( m_1,m_2,....,m_t)$ *under* $p_k$ *where* $C( m_1,m_2,....,m_t)$ *is output of* *C* *on inputs* $(m_1,m_2,....,m_t)$. Consider a scenario with respect to healthcare where a doctor or patient send sensitive information to cloud for predicting some results as illustrated in Fig. 1.

- Step 1: Client (Patient or Doctor) sends data to third party service provider for performing a query/operation. Before sending, client encrypts the data.
- Step 2: The client then asks the cloud service provider to perform a certain function on the data and provide the results.
- Step 3: Cloud service provider then performs operations using homomorphic encryption property with some function f(). *Noise is produced with arbitrary large number of operations. Every addition operation adds to the noise, and every multiplication operation double the noise. High computation on encrypted data results in excessive noise and there are several noise reduction methods. One of the most popular methods are bootstrapping and squashing. Both of these methods are a self-reliant process that progresses without external help. Modulus switching is another prominent strategy for achieving better asymptotic performance than bootstrapping.*
- Step 4: Encrypted results are provided to client.
- Step 5: Client at its end computes decryption using decryption function and recovers f(message).
  $$\begin{aligned} Dec(Enc(f(message)))=f(message) \end{aligned}$$
  As a result no information is lost by getting encrypted results and decryption at its own end.

### Motivation

Security is the key concern in cloud computing. Data provided to third-party service providers for processing and automation are a big issue. Every single business or organization wishes for the personal and sensitive data of the user. Every organization whether government, private, healthcare, academia requires data for policy formation, research purposes, planning new marketing strategies, or launching innovative products. Our main concern is with healthcare privacy as according to a survey by acumen research and consulting [1] healthcare cloud computing market will pass US$ 40 billion by the year 2026. Cloud computing in health care not only increases efficiency but also reduces cost. It is fast but protection of patient-sensitive data is the prime concern as it will not only promote patient confidence but also help in economic development. The digitization of a patient’s medical records is supposed to improve care quality and efficiency while reducing costs. On the other hand, patient records include a considerable quantity of sensitive information. As a result, patients must be able to swiftly allow a range of medical affiliates to access their sensitive information using a simple, trustworthy, efficient, and secure approach. Therefore, it is vital to look at the usage of homomorphic encryption in healthcare and compare various homomorphic algorithms for illness prediction as well as data querying while protecting the privacy of patient information.

The rest of this article is organized as follows: the next section contains review methods, planning, inclusion and exclusion criterion, research questions with motivation. The subsequent section compares and analyzes homomorphic encryption starting with partial and somewhat homomorphic encryption approaches followed by fully homomorphic encryption approaches. Techniques to fully homomorphic encryption were classified into four categories, with significant approaches in each category were compared. The next section focuses on homomorphic encryption in the healthcare sector. Homomorphic encryption techniques were compared on the basis of communication and computation overhead for securely identifying LQTC, cancer, average heart rate, cardiovascular problems, and a secure query generating system in healthcare. Finally, conclusion is presented.

## Review method

**Fig. 2:**
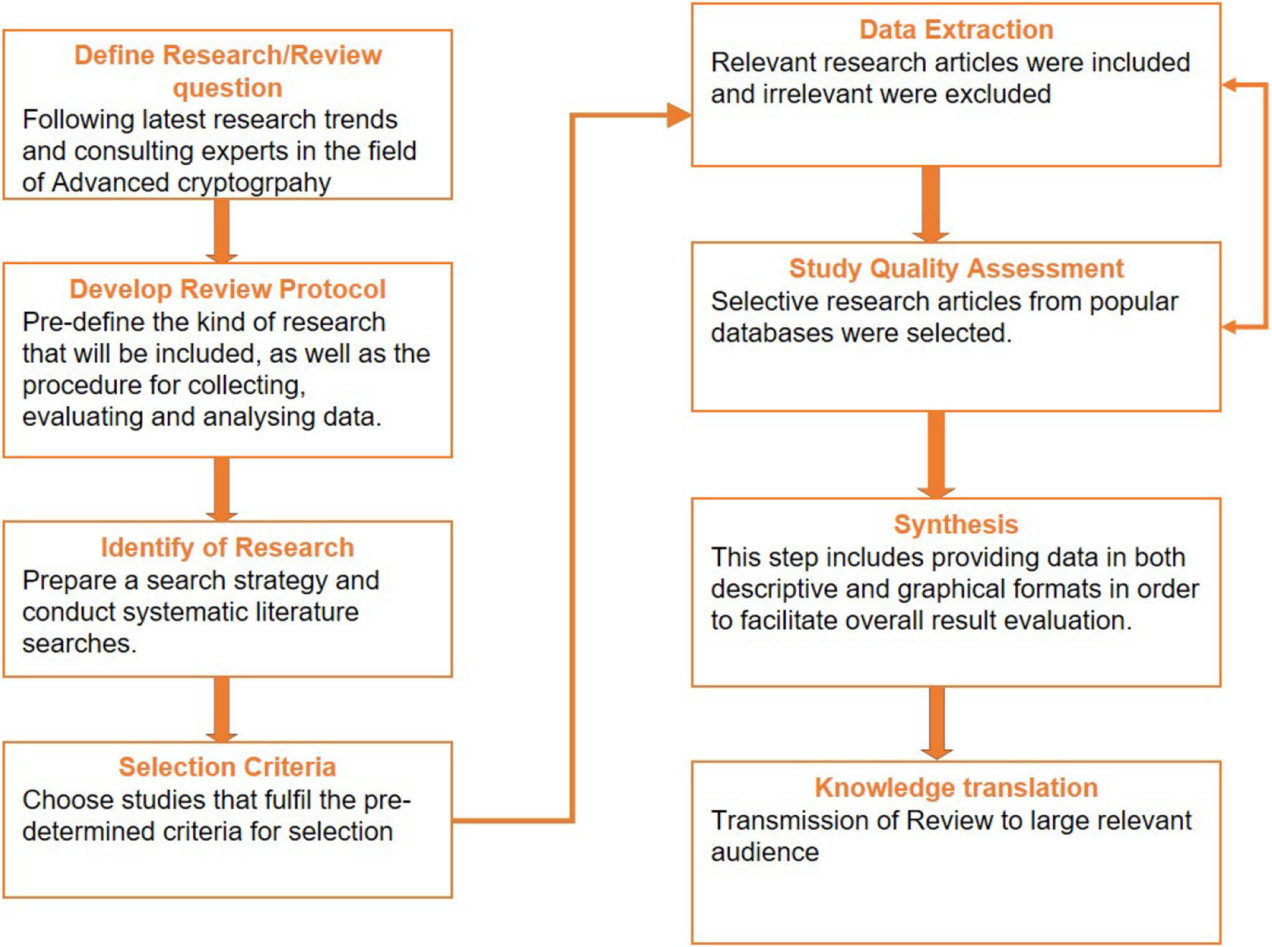
Steps in systematic review

The systematic review process may be considered as a means of solving a specific research problem. Presently no systematic reviews are focusing on homomorphic cryptosystems in healthcare and bioinformatics, therefore a systematic review research methodology was chosen. As a result, the systematic review aims to close this significant research gap. Kitchenham and Brereton [2] recommendations were selected to evaluate and explain all homomorphic encryption research questions. Our work is motivated by the revolutionary work of Craig Gentry [3, 4]. Additionally, the fact that fully homomorphic encryption will act as a boon to the healthcare industry as it will preserve complete privacy of patient health data is also a path of motivation. Reviewing the processes outlined in Fig. 2 will give a basic understanding of the systematic review process:

- ***Define research/review question:*** After reading various research/Journal articles and magazines and consulting with the expertise in the area of homomorphic encryption. Various research questions were identified.
- ***Develop Review Protocol:*** Pre-define the kind of research that will be included, as well as the procedures for collecting, evaluating, and analysing data.
- ***Identification of Research:*** After, Gentry’s revolutionary work in homomorphic encryption, a number of homomorphic encryption methodologies in healthcare and bioinformatics were published. Despite tremendous research in homomorphic encryption there is no systematic review that considers quality of research and development.
- ***Extraction:*** Relevant research articles were included and irrelevant were excluded.
- ***Study Quality Assessment:*** Research articles were selected from popular repositories with keywords as homomorphic encryption to review basic homomorphic approaches.
- ***Data Synthesis:*** This phase entailed presenting data in descriptive and graphical form. It will help to make the overall evaluation of outcomes easier.
- ***Knowledge Translation:*** The findings and details of the review will be distributed to relevant target groups in a variety of media.

### Review planning

The accomplishment behind each review depends on the selection of good-quality papers with unique work and genuine references. Thus, recognized journals, conference proceedings, and databases were investigated and explored in the area of homomorphic encryption. Scientific publications from Scopus, ACM Digital library, Springer Link, Sciencedirect, Google Scholar were selected based upon research questions given in Table 1. Fundamental studies in PHE, SWHE that function as cornerstones of homomorphic encryption were chosen first, followed by publications focused only on fully homomorphic encryption. A total of 1815 scientific papers were obtained using a database search with Keywords used for search as ’homomorphic encryption’, and ’homomorphic encryption’ or “medical” OR “healthcare” OR “bioinformatics” OR “EHR” OR “patient” OR “health” OR “medicine”. Following the removal of duplicate documents, a total of 857 records were evaluated for title screening. After screening title total 194 articles were selected for abstract screening. In abstract screening 69 papers that were focused on homomorphic encryption based upon LWE, NTRU, Lattices, Integers, and HE papers focused mainly on healthcare and bioinformatics were selected. Other than that 19 important papers in PHE and SWHE (consider Fig. 4) were chosen for better understanding the concept of homomorphic encryption.

**Fig. 3:**
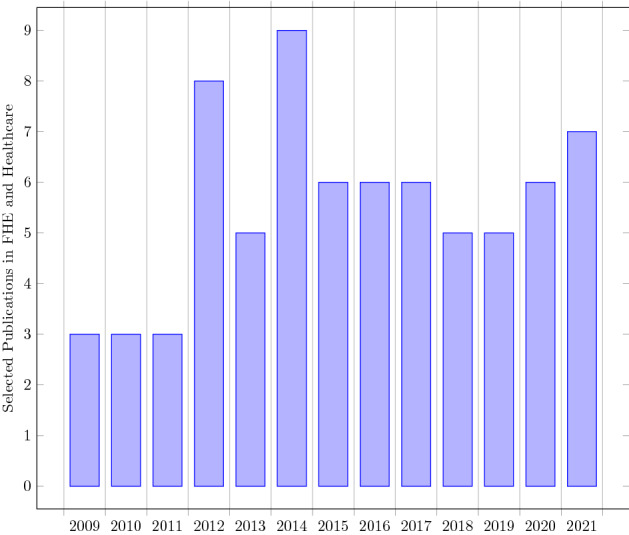
Scientific Articles from year 2009 to 2021

### Inclusion and exclusion criteria

Scientific articles in journals, conferences proceedings, workshops published in the year range from Jan 2009 to Dec 2021 (Fig. 3) were considered. Other than that, Partial and Somewhat homomorphic encryption papers of the old era were also included to better understand the concept of homomorphic encryption. All quality research publications of fully homomorphic encryption after the Gentry FHE scheme were considered. Articles that were focused on the applicability of homomorphic encryption other than healthcare or bioinformatics were excluded.

### Research questions

Prime objective of this review writing is to categorize the current literature on homomorphic encryption with its contributions in health informatics. This research study’s end result is the identification and examination of homomorphic encryption methods in healthcare. A set of research questions are formulated for this systematic literature review in Table 1.

**Table 1:** Research questions

Research Questions	Motivation
1. **Research Study in the area of partial homomorphic encryption.** 1. What are the various types of homomorphic approaches? 2. How to compare and analyze these approaches on the basis of homomorphic encryption and decryption methods used? 3. What hard problem is used that act as a base of cryptographic approach?	Homomorphic encryption techniques are built on the foundation of partial and somewhat homomorphic encryption. It’s crucial to review by comparing all types of homomorphic systems. These homomorphic encryption approaches are examined based upon hard problems, efficiency, and accuracy.
2. Revolutionary work in Homomorphic Encryption 1. Why Craig Gentry work is considered as revolutionary work in homomorphic encryption? 2. How somewhat homomorphic encryption act as fully homomorphic encryption using bootstrapping?	Craig Gentry provided the first FHE approach. The implementation of Gentry’s groundbreaking FHE method resulted in a slew of advancements that established the basis for a slew of subsequent projects. This development has been classified in a variety of ways. At first, the Gentry Scheme was somewhat homomorphic, but bootstrapping made it fully homomorphic. It is important to study and understand the Craig gentry approach as it leads to advancement in fully homomorphic encryption mainly in the area of machine learning [5] as machine learning is one of the most interesting applications and has drawn a lot of attention from researchers.
3. Homomorphic Encryption in Healthcare and medical Informatics 1. What are the various approaches of homomorphic encryption in healthcare and medical informatics? 2. How has homomorphic encryption in healthcare industry is evolved over time? 3. What evaluation methods have been used to check the correctness of these approaches?	Digitizing a patient’s health information is expected to increase care quality and efficacy while lowering costs. On the other hand, patient records include a great deal of sensitive information. As a result, a simple, trustworthy, efficient, and secure method is required in which patients may quickly permit a variety of medical affiliates to access their sensitive information. Therefore, it’s critical to investigate the use of homomorphic encryption in healthcare and evaluate different homomorphic methods for disease prediction, querying of data, while maintaining patient data privacy.

## Background

The medical services industry is experiencing a digital revolution. Modernizing medical care has prompted another time of computerized wellbeing and health. Medical services information is gathered from different sources (e.g., sensors associated with patients) and stored in unique medical services clouds (e.g., private and public clouds). Also, the volume of agglomerated medical information is sufficiently enormous to qualify as “Big Data”. As cloud medical services become a well-defined component in the medical services industry, there is a more critical requirement for safely sharing patient data across such dissimilar medical services clouds. Besides, with Accountable Treatment Organizations (ACOs) (e.g., medical service providers, specialists, clinics, and protection providers) collaborate to provide top-notch care, with demand for constant availability across cloud medical services higher than at any point in recent times. A disentangled patient-driven paradigm, in which patients can switch suppliers while still providing their data in a useful way for better diagnosis and treatment, and, in the long term, for enhanced global health, is appealing. As of now, medical care suppliers who have delicate patient information in private medical care clouds across the globe are reluctant to share that data on account of security and protection issues. As medical care suppliers move to the local area and public cloud-based administrations, a requirement for a secure connection between divergent medical care cloud increments. Moreover, security guidelines forced by Health Insurance Portability and Accountability Act (HIPAA) [6] and Health Information Technology for Economic and Clinical Health (HITECH) [7] place a cumbersome undertaking on medical care Information Technology (IT) framework to be agreeable with protection and security guidelines. Moreover, with arising Internet of Things (IoT) market and its mix in the vast information cloud stage, there is expanded worry about security and protection with the medical services cloud worldview. Many researchers contributed with their study in homomorphic encryption. Homomorphic encryption has three types: partial homomorphic encryption (PHE), somewhat homomorphic encryption (SWHE), and fully homomorphic encryption Fig. 5. PHE supports either addition or multiplication i.e. one operation over encrypted data. SWHE supports a finite number of operations, i.e., it evaluates the circuit up to some limit or depth. FHE supports both operations infinite number of times over encrypted data.

**Fig. 4:**
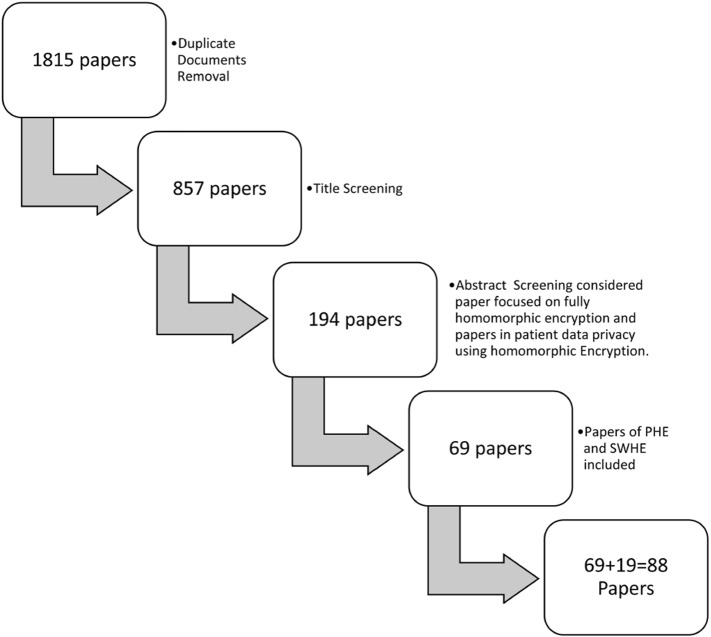
Research articles selection procedure

### Partial homomorphic encryption schemes

There are various partial homomorphic encryption schemes available. It all started by Rivest, Adleman and Dertouzos [8] with an asymmetric encrypted system that supports multiply operation over ciphertext and first time they uses the term “privacy homomorphism”. It was based upon hardness of prime factoring problem. Goldwasser-Micali [9] proposed first scheme with semantic security proof. It was based upon the hardness of quadratic residuosity assumption [10] which states that if $q\in Z_N ^*$, is *q* $\equiv$ $x^2 mod N$ where

$$\begin{aligned} Z_N ^*=\{y | 1\le y\le N-1\quad and\quad gcd(x,N)=1\} \end{aligned}$$

is solvable then q is *quadratic residue mod N* otherwise q is* quadratic non-residue N*. In, 1985 Tahar Elgamal [11] proposed a better public key cryptosystem which was an improvement over Diffie-Hellman[12] key exchange algorithm. In this public-key cryptography, security depends upon the hardness of discrete algorithms over finite fields. In discrete algorithm, consider a prime number *p*. let $\alpha$ and $\beta$ be integers which cannot be divided by *p* then find *x* such that $\alpha ^x$ $\equiv$ $\beta$ (*modp*). Dense probabilistic encryption by Benaloh [13] is a homomorphic encryption scheme over addition operator with an improved expansion factor as compared to Goldwasser-Micali. The proposal was based upon higher residuosity problem[14] $x^n$ as compared to quadratic residuosity problem, which is $x^n$ in GM cryptosystem. Benaloh approach has a homomorphic property which shows that encrypted text multiplicative operation is the same as plain text addition operation. As encryption was applied after messages were added, encrypted messages $Enc(m_1)$ and $Enc(m_2)$ may be directly assessed. As a result, one can deduce that Benaloh’s method was additive homomorphic. Paillier [15] in 1999 proposed a trapdoor approach compared to prime residuosity, Paillier approach was based upon composite residuosity classes, which believed to be kind of boon to public-key cryptosystems. Initially, the nature of Paillier cryptosystem allows addition operation over encrypted data but later improvements in the approach proved that multiplications can also be performed over encrypted data. There are other partial homomorphic schemes [16–18] that were based upon previous work by Elgamal, Benaloh and Paillier, which improves computational performance while maintaining homomorphic property. In [19], due to hardness of the lattice problems, the author, proposed homomorphic cryptosystem with addition operation over vast cyclic group. Authors called their proposed scheme’s homomorphic attribute *pseudohomomorphic*. Galbraith [20] proposed a more natural generalization of Paillier’s cryptography approach, which he applied to elliptic curves while keeping the Paillier’s cryptosystem’s additional homomorphic property. Various partial homomorphic encryption (Table 2) approaches are compared based upon encryption, decryption, hardness, and homomorphic property.

**Table 2:** Comparison of partial homomorphic encryption schemes

Name of algorithm	Additive homomorphic	Multiplicative homomorphic	Hard problem base	Key generation	Encryption	Decryption
[21]	No	Yes	Large prime factorization	$\phi$ =$(q-1) (p-1)$, *ed* $\equiv$ $1 mod\phi$*,q and p are prime nos. and (e,d) is key pair*	$c=m^e mod n$	$m=c^d mod n$
[9]	Yes	No	Quadratic residuosity	n=pq and random y as quadratic non residue modulo n i.e.. $$ (\frac{y}{n}=1) and (y,n) $$ as public key and (p,q) is secret key	$$E(m) = x^2y^m (mod n), where $$ $$m=\{0,1\}$$ and $$ gcd(x,n)=1$$	*Based upon cipher text if it is quadratic residue modulo n or not. if yes then return 0 otherwise 1*
[11]	No	Yes	Discrete logarithms	Public key is $$ (g,q,y_A)$$, $$y_A=g^a$$ in $$\mathbb {Z}^*_p$$ and Private key is *a*, *p* being large prime, *g* being a cyclic group’s generating element $$\mathbb {Z} ^*_p$$ and $$q=p-1$$, a is any random number.	$$(c_1,c_2)=(g^k,my_A^K)$$ in $$\mathbb {Z}^*_P $$	$$c_2(c_1^a)^{-1}$$ in $$\mathbb {Z}^*_P $$
[13]	Yes	No	High residuosity problem	Two large prime numbers with a block size *r* and *p* and *q* with: *r* divisible by $p-1$, *r* and $p-1/r$ should be relatively prime, *r* and $q-1/r$ being relatively prime and $n=pq$. Choose *y* $\in$ $(\mathbb {Z}_n)^*$ $=\{ x \in \mathbb {Z}_n:$ gcd$(x,n)=1$} such that $y^{\varphi /r}$ $\ne$ 1mod*n*, $\varphi$ is $(p-1)(q-1)$, public key is *y*, *r*, *n* and private key is *p* and *q*	$E_r(m_1)$=$\{ y^{m_1}u^r$ mod $n: u \in$ $(\mathbb {Z}_n)^*\}$, Where $m_1$ is an element in $\mathbb {Z}_r$ and *u* a random number in $(\mathbb {Z}_n)^*$	$(y^{-i}c)^{\varphi /r} \equiv 1$ message $m_1$ is generated by extensive search for *i* $\in$ $\mathbb {Z}_r$
[15]	Yes	No	Composite residuosity problem	*p* and *q* being large prime numbers such that $gcd ((p-1) (q-1),pq)=1$. calculate $n=pq$ and $\lambda$ (*carmichael’s function*)=$lcm(p-1,q-1)$, select random integer *g* $\in$ $\mathbb {Z}^*_{n^2}$with $gcd(n,L(q^{\lambda mod n^2}=1))$ where function *L* *(Lagrange function)* $=L(u)'=\frac{(u-1)}{n}$ for every u within subgroup $\mathbb {Z}^*_{n^2}$ Public Key (Encryption): (*n*, *g*) Private Key (Decryption): $\lambda$	$E_m=g^m \cdot r^n mod n^2$ where $m<n$	$M=D(E_m)=\frac{L(E_m^\lambda (mod n^2))}{L(g^\lambda (mod n^2))}mod n$
**Name of Algorithm**	**Homomorphic property**					
[21]	$$Enc(m_1)*Enc(m_2) =(m_1^e (mod n))*(m_2^e(mod n)) =(m_1*m_2)^e (mod n) =E(m_1*m_2) $$					
[9]	$$Enc(m_1)*Enc(m_2)=(x_1^2y^{m_1} (mod n)) *(x^2_2y^{m_2}(mod n))=(x_1*x_2)^2y^{m_1 + m_2}(mod n)= E(m_1+m_2)$$					
[11]	$$Enc(m_1)*Enc(m_2) =(g^{k_1},m_1y_A^{k_1})* (g^{k_2},m_2y_A^{k_2})=g^{K_1 +K_2},m_1*m_2y_A^{K_1+K_2}=E(m_1*m_2)$$					
[13]	$$Enc(m_1)*Enc(m_2)=(y^{m_1}u_1^r(mod n)) * (y^{m_2}u_2^r(mod n))=y^{m_1+m_2}(u_1*u_2)^r (mod n) =E(m_1 +m_2 (mod n))$$					
[15]	$$Enc(m_1)*Enc(m_2)=(g^{m_1}{r_1}^n (mod n^2))*(g^{m_2}{r_2}^n (mod n^2))=g^{m_1 +m_2}(r_1*r_2)^n mod(n^2)=E(m_1+m_2)$$					

### Somewhat homomorphic encryption schemes

SWHE schemes [22–26] support an arbitrary number of operations, but with each operation, a noise is generated and after a limit the underlying encrypted value is lost. As a result, SWHE systems support arbitrary operations but only for a finite number of times. Each addition operation increases noise and every multiplication operation causes noise to multiply. SWHE is a unique but not complete solution like fully homomorphic encryption. FHE supports an unlimited number of arbitrary operations over ciphertext. Boneh-Goh-Nissim [23] proposed a semantically safe technique for adding and multiplying encrypted data. It supports an arbitrary number of addition with a single multiplication operation on ciphertext with a specified length. BGN enables evaluation of quadratic formulas over ciphertexts, i.e., 2-DNF (*Disjunctive Normal Form*). BGN approach hardness was dependent upon the subgroup decision problem [27], which states that whether a given element $g_1$ of a finite group *G* is a member of subgroup $G_1$. Yao [22] study is an early effort for performing functions operations on the ciphertext. As a solution to the Millionaires’ Problem, Yao developed a two-party communication protocol, which compares the wealth of two wealthy persons without disclosing the precise amount.

Furthermore, in this approach, the ciphertext size grows linearly at a minimum as every gate in the circuit is computed. Yuval Ishai and Anat Paskin (IP) [24] proposed a scheme based upon a branching program evaluation on encrypted data. In [26] author proposed first SWHE scheme over a semi-group and NC1 is a complexity class covering circuits with poly-logarithmic depth and polynomial dimension. With one OR/NOT gate, the suggested technique allowed for polynomially many ANDing of ciphertexts. With each multiplication size of ciphertext increases.

**BGN scheme**

*Key generation:*

Consider $$C_G$$ and $$C_{G_1}$$ as multiplicative cyclic groups of order *n* with $$n=q_1q_2$$. let *g* be a generator of $$C_G$$ with $$e:C_G \times C_G \rightarrow C_{G_1}$$ is a bilinear map, public key is $$ (n,C_G,C_{G_1},e,g,h)$$ with *a* as generator of $$C_G$$ $$g,a\xleftarrow {\text {R}}$$ and set $$h=a^{q_2}h$$ being random generator of subgroup of $$C_G$$ of order $$q_1$$.

*Encryption:*

$c_{p1}=E_{msg_1}=g^{msg_1}h^r mod n$, where *r* being random number $\in$ {0,1,.,.,.n-1}.

*Decryption:*

$$msg_1=D(c_{p1})=\log _{g'}c'$$

*Homomorphic Property under addition:*

$$\begin{aligned}&={c_{p1}c_{p2}h^r}\\&={g^{msg_1}h^{r_1}g^{msg_2}h^{r_2}h^r}\\&={g^{msg_1+msg_2}h_1^{r'}} \end{aligned}$$

Where, $$r_1+r_2+r=r$$

*Homomorphic property under multiplication:* Consider $g_1$ and $h_1$ with order *n* and $q_1$, respectively. set $g_1=e(g,g)$ and $h_1=e(g,h)$. Also consider $h=g^{\alpha q_2}$, $\alpha \in \mathbb {Z}$.

$$\begin{aligned} c&={e(c_{p1},c_{p2})h_1^r}\\&={e(g^{msg_1}h^{r_1},g^{msg_2}h^{r_2})h_1^r}\\&={g_1^{msg_1msg_2}h_1^{msg_1r_2+r_2msg_1+\alpha q_2r_1r_2+r}}\\&={g_1^{msg_1msg_2}h_1^{r'}} \end{aligned}$$

with $r'=msg_1r_2+r_2msg_1+\alpha q_2r_1r_2+r$,uniformly distributed in $\mathbb {Z}$ and ciphertext is uniformly distributed in $C_{G_1}$ instead of $C_G$.

### Fully homomorphic encryption schemes

An encryption scheme is fully homomorphic if it supports two operations, i.e., addition and multiplication, an infinite number of times over encrypted data. Ciphertext contains noise that increases with operations in all the fully homomorphic encryption schemes. In particular, a multiplication operation increases more noise than an addition operation. The noise level gets too high at a certain threshold, preventing additional homomorphic procedures from resulting in accuracy. At this point, a refreshing procedure is required, which makes the performance highly unsatisfactory.

#### Preliminaries for FHE

**Fig. 5:**
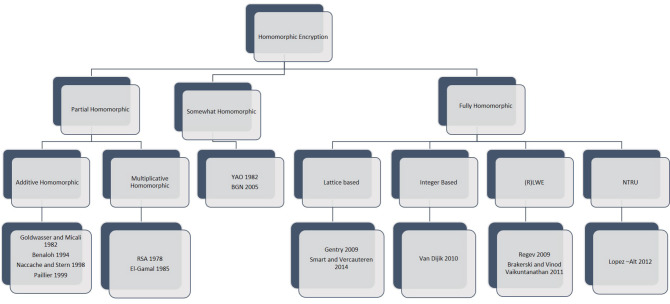
Classification of homomorphic encryption

In this section, the FHE scheme is classified into four categories (Fig. 5). These advanced cryptographic approaches act as preliminaries for fully homomorphic encryption schemes. A review of research papers based on these criteria is also conducted.

- Lattice based cryptography
- Learning with errors (LWE/RLWE)
- Integer based
- NTRU

***Lattice-based cryptography***

Lattice-based cryptography is an advanced cryptosystem. It holds first place in the advanced post-quantum cryptosystem, i.e., Cryptography based on lattices is thought to be safe against superfast quantum computers. Lattice-based cryptosystems are based upon hard problems like *Shortest Vector Problem* to find the shortest non-zero vector in the lat and *Closest Vector Problem* with the goal of finding the closest lattice vector with the given vector. * In n-dimensional space, a lattice is any regularly spaced grid of points. Formally,n independent vectors known as basis of the lattice* $b_1,b_2,b_3,\dots$,$b_n$ *are generated as a set of vectors *

$$\mathcal {L}$$($$b_1,b_2,b_3,\dots ,b_n)= \sum _{j=1}^{n} x_jb_j $$, $$x_j \in \mathbb {Z}$$.

In 2009 Gentry [3] proposed a fully homomorphic encryption scheme, which was the first fully homomorphic encryption scheme. The solution comprises three steps. In the *first* step it provides general result that can evaluate its own decryption circuit i.e. *bootstrappable *. In the *Second* step, a public key encryption approach was suggested. This approach uses the concept of ideal lattices with having a bootstrappable property. It is represented as the lattice FHE technique that allows both homomorphic addition and multiplication computations on encrypted data. Former HE approaches provide either of the two operations as in Table 2; therefore, those schemes were partial homomorphic. The challenge of FHE is noise which increases as number of homomorphic operations increase. If the noise becomes too large, it becomes challenging to decrypt correctly.

Gentry’s approach is *somewhat homomorphic (SHE)* which means it can handle homomorphic operations up to a limit and to make it for unlimited operations ciphertext needs to be refreshed using *bootstraping* procedure. This procedure is a recrypting process, and inefficient. Initially, the encryption was bitwise, meaning that each bit of data was encrypted independently, and the result was ciphertext. Bit addition and multiplication (modulo 2) correspond to XOR and AND bitwise operations, allowing any Boolean circuit to be evaluated, i.e., any calculation to be done by first defining the computation in XOR and AND gates. However, immediately breaking down a calculation into bit operations results in complex and deep computation circuits, which SWHE cannot handle and requires encryption, i.e., Bootstrapping.

***Bootstrapping and squashing***

If a scheme is homomorphic enough to evaluate its circuit of decryption, it can be transformed into fully homomorphic encryption capable of evaluating any function. High computation on encrypted data results in high noise. It is necessary to refresh encrypted data regularly, but this must be done without needing a secret key, which is accomplished by bootstrapping. Bootstrapping is a self-reliant process that progresses without external help. Suppose SWHE can handle a circuit up to a certain threshold depth, *D*. If the augmented decrypt circuit has depth $\le D$, then the system is bootstrappable, i.e., the ciphertext can be refreshed through recryption. The idea behind recryption is:

Consider two secret key pairs. $(sk_1,pk_1)$ and $(sk_2,pk_2)$ and $Decrypt_E(sk_1, Encrypt_E(pk_1,m))=m$ for message say *m* and same for second pair. Suppose *E* is homomorphic with respect to decryption circuit. Encryption of $sk_1$under $pk_2$ will be $Encrypt_E(pk_2,sk_1)$. Encryption of initial ciphertext with public key

$$pk_2:$$ $$Encrypt_E(pk_2, Encrypt_E(pk_1,m))$$

Consider

$$Decrypt_E(Encrypt_E(pk_2,sk_1),Encrypt_E(pk_1,m))$$ $$=Encrypt_E(pk_2,m)$$

and it is to be done bitwise. In this way inner encryption is removed by creating a newly encrypted ciphertext with $pk_2$ and the scheme can homomorphically evaluated.

$$Decrypt_E(sk,c_1) + Decrypt_E(sk,c_2)$$ or,

$$Decrypt_E(sk,c_1) * Decrypt_E(sk,c_2)$$.

Bootstrapping is valid in decryption algorithms with lesser circuit depths. But if the circuit depth is high, bootstrapping is not possible due to performance issues. As a result, an improved technique is applied known as *squashing*, which reduces decryption algorithm complexity. According to this scheme, initially, a set of vectors are selected. The sum of these vectors is the same as the multiplicative inverse of the private key. Set elements are then multiplied with the encrypted text. As a result, polynomial degree of the circuit is reduced to an acceptable level. Retrieving private keys depends upon the assumption of SSSP (Sparse Subset Sum Problem) mentioned in [28]. Craig Gentry and Shai Halevi [29] proposed an FHE scheme that is hybrid of SWHE and MHE, i.e., Multiplicative homomorphic encryption. The approach still depends upon bootstrapping step, but it avoids squashing as it does not rely on the assumed hardness of SSSP. A new method in which sparse subset sum problem was replaced with Decision-Diffie Hellman [30]. This approach additionally substitutes MHE with AHE (additively homomorphic encryption), which encrypts discrete logarithms and creates a leveled FHE whose difficulty is determined by the shortest independent vector problem over ideal lattices [31]. At PKC 2010, a modified Gentry’s fully homomorphic public-key encryption scheme was presented by Smart and Vercauteren [32] that can support SIMD style operations. Gentry and Halevi followed the slow key generation process of smart and Vercauteren but still lacks SIMD style operation. A realization of achieving Single instruction multiple data operations using homomorphic evaluation of AES was given by smart and Vercauteren in 2011. BDDD, i.e., bounded distance decoding problem for somewhat homomorphic, ensured the encryption scheme’s security and sparse subset sum problem for bootstrapping procedure.

***Integer-based FHE schemes***

A SWHE was presented [33] based upon integers with the hardness of algorithm depending upon *Approximate Greatest Common Divisor* problem [34]. Although the scheme was symmetric, It was also demonstrated basic symmetric homomorphic encryption technique that may be transformed to an asymmetric homomorphic encryption technique [33]. At Eurocrypt 2013, an extended version of fully homomorphic encryption over integers was presented with batch FHE scheme over integers [35].Using the renowned Chinese Remainder Theorem, the DGHV method was extended by packing *l* plaintexts $m_0,m_1,\cdots ,m_l$ into a single ciphertext. It allows not only encrypting bits but also elements from rings of the form $\mathbb {Z}_Q$. There are also some additional FHE schemes proposed over integers. A novel FHE over integers that is scale-invariant [36], an approach with integers plaintext [37], a symmetric FHE scheme that does not require bootstrapping [38], a SWHE method for performing arithmetic operations on huge integer values without converting them to bits [39]. In their names imply, all of these approaches enhanced FHEs over integers.

***Learning with errors***

Learning with errors (LWE) is a key concept that serves as a building block of advanced cryptographic algorithms. LWE is a generalization of LPN (*Learning parity with noise*) problem. LWE can be thought of as two sub-problems closely related to each other. *Search LWE* and *Decision LWE*. LWE is a powerful cryptography tool as its hardness is the same as lattice problems, i.e., shortest vector problem and closest vector problem.

*Consider a matrix with* *n* *rows and* *m* *columns with uniform random entries from space of integers modulo* *q*. *let* *Z* *be a vector of length* *n* *with uniform random entries from the same space and let* *b* *be a vector of length* *m* *with entries drawn from* $\chi$ *distribution. Compute* *s* *(all arithmetic operations are performed mod* *q*)

$$a^TZ + b^T =s^T$$

In [40] the hardness of worst-case lattice issues was decreased from SVP to LWE problems, implying that if an algorithm can solve the LWE problem in an acceptable period, it can also solve the SVP problem. In [41] a significant improvement to the LWE problem by developing the ring-LWE (RLWE) problem was proposed, which leads to new applications. It was also proved that RLWE issues could be reduced to worst-case problems on ideal lattices, which polynomial-time quantum algorithms struggle to do. Another practical RLWE [42] to obtain an FHE scheme was proposed which, employs Gentry’s squashing and bootstrapping techniques. Polynomial-LWE (PLWE), a simpler variant of RLWE, was employed. PLWE can likewise be reduced to worst-case scenarios like SVP on ideal lattices. To reduce the noise growth concerning each operation, Brakerski, Gentry, and Vaikuntanathan introduced a leveled homomorphic encryption approach [43]. The growth of the noise is linear in contrast with the multiplicative depth of the circuit. The scheme was based upon a modulus switching technique known as the BGV scheme. BGV scheme was also used with AES to perform homomorphically in [44], but it requires different versions of public key, thus requiring a large amount of memory machine. Brakerski proposed a new approach invariance, at Crypto 2012 for leveled for leveled homomorphic encryption schemes. In contrast to modulus switching, ciphertext retains the same modulus throughout the homomorphic evaluation. As s result, just a single copy of the scale-invariant evaluation key must be maintained. In [46] Brakerski’s FHE scheme transformed from LWE to RLWE with detailed analysis of various subroutines, including multiplication, bootstrapping, and relinearization. No implementation of this scheme is available, but there is a proof-of-concept execution in computer algebra system which is used in [47]. In [48], a *relinearization* technique was introduced that obtains a SWHE without requiring hardness assumption on ideals. A *dimensional-modulus reduction* was also presented with the goal of converting *SWHE* to *FHE* without using the *squashing* and *SSSP*. Other schemes are based upon *Learning with Error problem*. In previous LWE schemes multiplication step was complicated and expensive due to relinearization but in [49] author suggested a novel approach for FHE, i.e., *approximate eigenvector* method. All addition and matrix operations in this approach are through matrices resulting in an asymptotically faster approach and easier to understand. The relinearization step was eliminated, and matrices were added and multiplied simply.

***NTRU-based FHE scheme***

NTRU-based encryption is an old encryption technique proposed by [50] in 1998. NTRU was the earliest encryption attempt on lattice problems. In [51] author uses on-the-fly MPC in which each user is involved in sending encrypted data to the cloud and decrypting, i.e., when outputs are received. A new type of encryption was used, i.e., *multikey* FHE with the capability of operating on inputs that are encrypted under multiple different and unrelated keys. The multikey FHE scheme was based upon NTRU, an efficient public-key encryption scheme. Although initially, NTRU was not fully homomorphic, transforming it into fully homomorphic reduces its efficiency but increases its capability. However, an additional assumption related to public key uniformity is needed, known as decisional small polynomial ratio (DSPR) assumption. This additional assumption is avoided by Stehle and Steinfield [52] and a secured FHE scheme was proposed [53] under RLWE with circular security assumptions only. A FHE technique was proposed based on the power-of-prime cyclotomic [54] ring. The method is based on the RLWE assumption does not need the use of the DSPR *(decision small polynomial ratio)* assumption. The approach’s efficacy leads to improved noise control, resulting in improved ciphertext storage, computation, and communication. In table 3, important FHE schemes based on their category and hard problem used are classified.

**Table 3:** Fully homomorphic encryption schemes

Name of algorithm	Lattice based	Integer based	(R)LWE	NTRU	Hard problem base
[3]	$\checkmark$				Bounded distance decoding problem over ideal lattices and sparse subset sum problem for squashing the decryption circuit
[33]		$\checkmark$			Approximate greatest common divisor
[43]			$\checkmark$		Ring based learning with error
[51]			$\checkmark$	$\checkmark$	RLWE and Decisional Small polynomial Ratio (DSPR) Assumption
[42]			$\checkmark$		SWHE uses hardness of RLWE and Squashing step uses SSSP (Sparse subset sum problem)
[29]	$\checkmark$				Shortest Independent Vector Problem
[32]	$\checkmark$				Bounded distance decoding problem for SWHE, Sparse subset sum problem for bootstrapping
[35]		$\checkmark$			Decisional approximate-GCD problem and error-free approximate GCD
[45]			$\checkmark$		GapSVP
[36]		$\checkmark$			Approximate-GCD problem
[49]			$\checkmark$		Approximate Eigen vector method

### Homomorphic encryption libraries

Research in HE has resulted in effective, freely accessible HE libraries and software. Custom implementations require a high level of programming and cryptography skill since noise should be carefully controlled to preserve accuracy upon decryption, and security settings must be explicitly specified. Table 4 gives an overview of the implementations, including the software’s name, inventor, language, license, and a brief synopsis of the schemes implemented.

**Table 4:** Fully homomorphic encryption in libraries

Library	Implemented algorithms	Summary
HElib [55]	[56, 57]	Helib is licensed under Apache 2.0 and created by IBM in C++. HElib also optimizes efficient homomorphic evaluation, with a focus on the Gentry-Halevi-Smart optimizations also using ciphertext packing techniques effectively
TFHE [58]	[59]	TFHE is a C/C++ package that provides gate-by-gate bootstrapping at a high speed. The utility allows us to evaluate any binary gated Boolean circuit over encrypted data without disclosing any data specifics
Seal [60]	[46, 57]	SEAL stands for Simple Encrypted Arithmetic Library is an open-source platform software library developed by Microsoft cryptography and privacy research group. Microsoft SEAL is an MIT-licensed developed in C++ that is simple to compile and run
FHEW [61]	[62]	FHEW is free software provided under the terms of the GNU General Public License. The library is written in the C programming language. The library utilises a symmetric encryption method to encrypt (and decrypt) single bit communications and allows evaluation of arbitrary Boolean circuits homomorphically over encrypted data using a public (evaluation) key.
HEAAN [63]	[57]	HEAAN is a software library that enables fixed point arithmetic with homomorphic encryption (HE). This C++ library allows you to perform approximate operations on rational numbers. The approximate error is affected by a number of factors, and the same is true for floating point operation errors
NFLIB [64]	[46]	The homomorphic encryption algorithm is implemented by FV-NFLlib, a software library (HE). FV-NFLlib is built on the NFLlib C++ library for ideal lattice cryptography and implements the Fan-Vercauteren (FV) scheme
PALISADE [65]	[56] [57, 62] [46, 59]	PALISADE implements lattice cryptography building blocks and popular homomorphic encryption approaches in an optimized way
CINGULTA[66]	[46, 59]	Cingulata is a C++ compiler toolchain and RTE that uses fully homomorphic encryption approaches to run C++ programmes over encrypted data
Lattigo [67]	[46, 57]	Lattigo is a Go module that implements homomorphic encryption primitives based on Ring-Learning-With-Errors and secure protocols based on Multiparty-Homomorphic-Encryption
Pyfhel [68]	Microsoft Seal, HElib, Palisade	Perform encrypted homomorphic computations such as addition, multiplication, scalar product, or matrix multiplication in Python with NumPy compatibility using PYthon For Homomorphic Encryption Libraries. Backends are SEAL/PALISADE
$\wedge$ $\circ$ $\lambda$ [69]	[56]	$\wedge$ $\circ$ $\lambda$ (Lol) is a general-purpose ring-based lattice cryptography library
cuYashe [70]	[53]	cuYASHE is the first GPGPUs implementation of the YASHE levelled fully homomorphic technique. To achieve significant speed increases, this library uses the CUDA platform and several algebraic techniques such as CRT, FFT, and polynomial and modular reduction optimizations

This section covers all homomorphic approaches beginning from PHE followed by SHE and FHE approaches. All these approaches based upon various factors like hard problems, nature of homomorphism are compared. FHE systems are also categorized, and they have improved and changed through time. Finally, the technique and programming language utilised in HE-implemented libraries are compared. The publications on homomorphic encryption that are explicitly focused on healthcare data are explored in the next section.

## Homomorphic encryption in healthcare

In the healthcare industry, patient health records are referred to as Electronic health records (EHR), a digital record of a patient’s medical history kept by hospitals or health care providers. It includes clinical data, demographics, medical history, and medical reports. Electronic health records (EHRs) are official health information records generated by any healthcare provider. Digitizing patients’ health care information is likely to improve the quality of care and effectiveness and reduce costs. It also facilitate the access of patients’ healthcare records whenever and wherever possible. The main goal of EHR’s is to reduce the communication gap between health care providers for a better quality cure and that too with reduced costs. However, Electronic health records (EHRs) contain a large amount of sensitive data, which is not only accessible to the doctor but also accessible to several different sources like insurance companies and staff members, so effective management of electronic health records is essential for concerns related to data security. The three fundamental and main goals of security is (CIA), i.e., Confidentiality, Integrity, and availability.

- Confidentiality: Only an authorized person should have access to the information.
- Integrity: Information should be correct, and an unauthorized person should not alter it.
- Availability: Information should be accessible, available, and usable at any time but only by an authorized entity.

A shared EHR comprises a complex and challenging layout of sensitive data, including laboratory tests, patient demographic information, medical history, radiology records like ECG, X-RAYS, and CTs. There is a requirement for a complete secured model that should meet the terms and conditions of legal and regulatory bodies. A secure model ensures access to sensitive information limited only to those entities who have a justifiable need-to-know privilege assigned by the patient. Sometimes, a patient chooses to deliberately hide his health information related to an HIV/AIDS treatment unless and until a specific treatment alternative is included. Therefore, it is necessary to have a simple, reliable, efficient, and secure mechanism in which patients can quickly authorize the number of medical affiliates for accessing their full or partial sensitive information. There are various techniques for data protection, including pseudonymisation and anonymization. Both of these techniques can be used to protect the individual’s health record.

***Anonymisation*** is the processing of data to prevent identifying the person to whom it belongs permanently. Anonymized data never allow the identification of an individual to whom it belongs. It is not even possible that any person could be known from the data by any advanced processing of the data. On the other hand, ***Pseudonymisation*** replaces any identifiable characteristics of data with a pseudonym. The only difference between pseudonymization and anonymization techniques is that it is possible that an individual can be identified by analyzing the related data in pseudonymization. In various cases, it is not possible to use an anonymization technique either because of the nature of data perspective. Pseudonymized data are very much suitable for processing and analyzing. However, anonymizing data while maintaining its value is a complex undertaking due to the inclusion of quasi-identifier components other than direct identifiers (e.g., name, address) that may be used alone or in conjunction with other data to re-identify it. The most common problems associated with Electronic health records are privacy, security, and confidentiality. Privacy of digital health records can be achieved in three different ways: ***Acquisition***, ***Storage*** and ***Computation***. Although first two are achievable, but the third condition, i.e. computation, also needs to be addressed to get complete privacy. There exists no system to enable processing in the cloud with privacy. FHE permits computation on encrypted data in the cloud. Various approaches satisfy homomorphic property on medical data. Multiple approaches are studied from 2009 till 2021 and focused on preserving patient privacy using homomorphic encryption. All of these approaches will be presented in the remaining parts of this section.

A decentralized system was proposed in [71] that authenticate users using verifiable attributes while maintaining attribute and identity privacy. Furthermore, an authentication system was designed with progressive privacy constraints in diverse interactions among involved entities. In [72]author has described a cloud-based approach using FHE to review ECG. Authors formulated FHE as the core of this idea and identified challenges to make it possible for ECG-Monitoring. Data obtained from THEW [73] standard ECG database that contains data of 24 hrs and took average heart rate calculation. Acquisition devices are capable of transferring data in AES format wirelessly. Encrypted data will only be stored in the cloud. However, to operate on some of the data, AES encrypted data first must be converted into FHE encrypted format. AES to FHE conversion done by the method described in [44]. Implementation was done with two algorithms one was gentry’s original [3] FHE scheme, and the other was BGV [43] scheme. In gentry scheme, recryption operation was costly and took $99.9 \%$ more time than homomorphic addition and multiplication. In BGV implementation, recrypt operation is avoided by defining multiplicative depth.

In [74], author presented an approach that predicts heart attack probability based upon few body measurements. They use a client application that collects health data and sends it in encrypted form and used Microsoft Windows Azure cloud service. A practical variant of a homomorphic encryption scheme was used. The method works with a ring where *n* is a power of 2, implying that polynomials with integer coefficients of degree less than *N* are the objects.

*R*= $$\frac{\mathbb {Z}[\,X\,]}{X^n+1}$$

Specifically plaintexts as well as ciphertext are all polynomials. An element $x \in R$ has the form

$$x= \sum _{i=0}^{n-1}x_iX^i$$, $$x_i \in \mathbb {Z}.$$

Key generation and encryption process uses two probability distributions $\chi _{key}$ and $\chi _{err}$ on *R*, where $\chi _{key}$ is used in key generation, i.e. uniformly distributed over polynomials with {-1,0,1} as coefficients in.

*KeyGen*$(N,q,t,\chi _{key},\chi _{err})$: With degree *N* and modulus *q* and *t* this algorithm samples small polynomials *f*’, *g* $\leftarrow$ $\chi _{key}$ and sets $f=[1+tf$’$]_q$ *if f is inverted modulo it computes* $f^{-1}$ of *f* $\in$ *R*, otherwise it chooses new *f*’ and set $h=[tgf^{-1}]_q$ and output public and private key pair as $(p_k,s_k)$=(*h*, *k*) $\in$ $R^2$.

*Encryption*(*h*, *m*): It samples small error polynomials $s,e \leftarrow \chi _{err}$. Cipher text generated is

$$c=[\lfloor {\frac{q}{t}\rfloor } [m]_t + e + hs]_q$$ $$\in $$ R

*Decryption*(*f*, *c*): With ciphertext *c* and private decryption key *f*, message *m* can be computed using decryption algorithm as:

$$m=[\lfloor \frac{t}{q}\cdot [fc]_q\rceil ]_t$$

In [75], FHE encrypted ECG data are uploaded from a patient to the cloud for detecting LQTC (Long QT Syndrome). Cloud will receive two values, QT and RR. These values are encrypted homomorphically at the patient’s end. Initially, recordings are taken from THEW database [73], and then patient $QT_c$ is computed homomorphically as

$$QT_c = \frac{QT}{\sqrt{\frac{RR}{sec}}}$$

$QT_c$ values are then compared with threshold value of 500*ms*.

$$f(QT,RR)=[QT^3 > (500)^3 (RR/sec)]$$.

After converting this equation into matrix, its performance is evaluated by comparing the Naive and Matrix approach results and found that the matrix approach is consistently more efficient than naive approach by a factor of 20. As in the naive approach, the depth of computation is higher, and cost increases with depth.

A secure computation [76] in personalized medicine using Paillier cryptosystem [15] was proposed, which allows a personal mobile device with sensitive data to compute tests (e.g., Warfarin dosage) while keeping personal data and test specifics private. Details of a partial implementation of this protocol were also revealed. In [77] author used the BGV [43] scheme and its practical implementation HElib [55] for calculating average heart rate, calculation of minimum and maximum heart rate, and detection of LQTS (Long QT Syndrome). As BGV scheme uses *leveled FHE* which eliminates expensive *recryption* operation. Leveled FHE uses a better technique for handline noise, i.e., *modulus switching*. In this scheme, plain texts are represented as polynomial rings $GF(p^d$ with *p* as a prime number, i.e., the range of polynomial coefficients and degree *d* of the polynomial. As plaint text are represented with polynomial rings which allow multiple message packing by partitioning them into independent slots [32] resulting into SIMD *Single Instruction Multiple Data* fashions.

The authors [78] developed evaluation techniques to compute securely over minor allele frequencies (MAF) and the $\chi$ statistic in genome-wide association studies. It was also explained how to calculate the Hamming distance and Edit distance between encrypted DNA sequences safely. Finally, the performance of two widely used homomorphic encryption algorithms [44] and [53] were examined. Another approach [79] proposed a data division homomorphic encryption scheme in wireless sensor networks. The algorithm divides whole data into two or three parts for safer communication and data storage. Forwarding nodes within the network upload encrypted sensor data without the actual need of decrypting it. In a worse case, if the forwarding node is attacked, the attacker will not eavesdrop original data. The algorithm is divided into five phases: data preprocessing, encryption, data transfer, data fusion, and decryption. Consider D as the sensed data and N as a random integer. The data division scheme will divide the data into *D*/*N*, *N*. It is both additive and multiplicative homomorphic as shown in equation 1 and 2.

$m=D/N$*or* $m=n$ *where* $m\in [0,M-1]$ *where M is large integer*

$$c=ENC(m,k,M)$$ *where*e $$k\in [0,M-1]$$

In the Data Transfer phase, encapsulation of packets take place with $\{E(D/N),k\}$*or* $\{E(N),k\}$, packets are then sent to forwarding nodes randomly and finally forwarding nodes stores and uploads the packet on the server. In the Data fusion phase integrity of the data is verified, based upon the number of packets received, i.e 2 for multiplication and 3 for addition central server fusion node fuse them with multiplication and addition and finally, in decryption phase central server, after fusion decrypt the data with *Dec*(*c*, *k*, *M*).

*Multiplicative homomorphism: due to homomorphism* *E*(*D*) *is the final result as:*

1 $$\begin{aligned}&{=Enc_1(D/N)*Enc_1(N)} {=Enc_1(D/N*N)}\nonumber \\&{=Enc_1(N)} \end{aligned}$$

*Additive homomorphism: due to homomorphism* $E2(D+M)$

2 $$\begin{aligned}&{{=Dec_1\{Enc_1\{Enc_2(D/N)\}*Enc_1(N)\}+Enc_2(m)}}\nonumber \\&{{=Dec_1\{Enc_1\{Enc_2(D/N)+Enc_2(D/N)+Enc_2(D/N)+.......Enc_2(D/N)\}}}\nonumber \\&{{+Enc_2(m)}}\nonumber \\&{{=Dec_1\{Enc_1\{Enc_2(D)\}+Enc_2(m)}}\nonumber \\&{{=Enc_2(D)+Enc_2(m)}}\nonumber \\&{{=Enc_2(D+m)}} \end{aligned}$$

In [80], author proposed a fully homomorphic encryption algorithm for encrypting and decrypting medical images. It uses DICOM as the input image. They analyzed keyspace analysis, correlation analysis, histogram analysis, key sensitivity analysis, *PSNR*, *MSE* and noise analysis. The author divided the whole process into fifteen steps. They used a probabilistic algorithm for a key generation as well as for encryption described in, and a deterministic algorithm for decryption [81]. This scheme supports unlimited additions (Eq. 4) and limited multiplications (Eq. 5) and uses a probabilistic algorithm for a key generation as well as for encryption and a deterministic algorithm for decryption. Perfect encrypted image results into uniform distributed histogram image according to histogram analysis of image pixel distribution was uniform. Larger PSNR values were identified, and in correlation analysis between two adjacent pixels, it was concluded that the algorithm provides good security. In key space analysis, $150-bit$ size key is considered, which has total $2^{150}$ values. Key size is sufficient, which is not affected by brute force attacks.

***Homomorphic property:***

$M_1$ and $M_2$ are plain texts and $C_1$ and $C_2$ ( equation3) as respective ciphertexts. Parameter $S_1$ and $S_2$ are two small positive integers and $B_1$ and $B_2$ are big random positive integers. *P* and *Q* be two big prime numbers with $P>>Q$.

3 $$\begin{aligned} C_1&=a^{S_1}(B_1Q+M_1)modP\nonumber \\ C_2&=a^{S_2}(B_2Q+M_2)modP \end{aligned}$$

*where a is any random number under P*

*Multiplicative Homomorphism:*

4 $$\begin{aligned}&{=a^{S_1}(B_1Q+M_1)modP*a^{S_2}(B_2Q+M_2)modP}\nonumber \\&{=a^{S_1 +S_2}(B_1B_2Q^2+B_1QM_2+M_1B_2Q+M_1*M_2)modP}\nonumber \\&{=a^{S_1 +S_2}((B_1B_2Q+B_1M_2+M_1B_2)Q+M_1*M_2)modP} \end{aligned}$$

*Additive homomorphism:*

5 $$\begin{aligned}&{=a^{S_1}(B_1Q+M_1)modP + a^{S_2}(B_2Q+M_2)modP}\nonumber \\&{=a^{S_1}(B_1+B_2)Q+M_1+M_2)modP(if S_1=S_2)} \end{aligned}$$

Another approach [82] performs privacy-preserving linkage of records in healthcare sectors. A secondary contribution of this research is the creation of an API for the El-Gamal cryptosystem [11] that enables for the use of the cryptosystem’s multiplicative homomorphic feature. In [83] a secure privacy-preserving data aggregation technique was developed using a Bi-linear ElGamal cryptosystem and aggregate signature. According to security analysis, the proposed system protects data confidentiality, authenticity, and privacy while also resisting passive eavesdropping and replay assaults from malicious adversaries. Because of the use of privacy homomorphism, this approach can be implemented in a cloud-assisted Wireless Body Area Network (WBAN). The [84] solution is built on the hybrid framework Chimera, which allows migration between distinct families of fully homomorphic schemes TFHE [58] and HEAAN [57]. It was the only bootstrapped solution in idash 2018 competition that may be used for a variety of parameters without requiring a recryption of the genomic database

In [85] author proposed and implemented an optimized NTRU-based homomorphic encryption scheme resulting in much slower growth of noise. The encryption scheme includes *d* as degree of number field, modulus *q*,$\sigma _k$ and $\sigma _c$ is standard deviation of discrete Gaussian error distribution in key and ciphertext space, $\lambda$ is the security level that can be 80 or 128 bits. There is a bit decomposition function *BD*() BD(*integer*) takes a $l-$ bit integer and output bit decomposition of integer as a row vector of size *l*. BD (*polynomial*) with input as *n* being polynomial size with *l* bit integer as each coefficient and outputs *l* $\times$ *n*. BD(*MatrixofPolynomials*) with input as polynomial matrix of size *x* $\times$ *y* and outputs a matrix of size *x* $\times$ *yl*. There is also bit decompose inverse function *BDI* which is an inverse of *BD*.

**Table 5:** Comparison of homomorphic encryption in Healthcare based upon disease prediction and performance

Paper	Database used	Disease prediction	Encryption time	Decryption time	Addition time	Multiplication time	Any other Processing Time	System Specifications
[72]	ECG Monitoring [73]	LTQC (Long QT Syndrome)	1.65 sec with BGV and 1.45 s with Gentry	0.65 s with BGV and 0.2 s with gentry	0.11 with BGV and $\le$ 1 s with gentry	0.8 in BGV and 1.79ms in Gentry	24.95 ms in gentry	dual-Xeon model E5450 node with 16 GB RAM, quad-cores frequency 3 GHz
[74]	Live data	Cardiovascular disease	13ms	11ms	< 1ms	39ms	-	Intel Core i7-3520 M at frequency 2893.484 MHz with polynomial degree 4096 and coefficient size 128 bit
[74]	Live data	Cardiovascular disease	577ms	549ms	1ms	5056ms	-	Intel Core i7-3520 M at frequency 2893.484 MHz with 16384 coefficients of size 512 bit
[77]	ECG Monitoring THEW [73]	LQTC (Long QT Syndrome)	0.41 s with ECG data interval of 24 hrs	0.31 s with ECG data interval of 24 hrs	–	-	–	Intel Xeon model E5-2695 processor with ram 252GB
[85]	–	Blood pressure	23ms with CPU and 0.16ms with GPU	5ms with CPU and 1ms with GPU	0.25ms with CPU and 0.07ms with GPU	87.8ms with CPU and 0.838ms with GPU	–	Intel Core-i7 5930K with frequency 3.5GHZ,DDR4 Ram 32 GB, GPU NVIDIA GeForce GTX980 with frequency 1126 MHz and 4GB memory
[86]	ECG monitoring THEW[73]	LQTS (Long QT Syndrome)	1.80 s and 0.26 s with bit length 32 and 16 respectively in 24 hr monitoring data	0.85 s and 0.25 s with bit length 32 and 16 respectively in 24 h monitoring data	502 min with bit length 32 and 1165 min with bit length 16 respectively in 24 hr monitoring data		–	Intel-Xeon workstation W3565 work with 4 cores and 8 threads, RAM 24 GB running 64-bit Ubuntu 15.04
[86]	ECG Monitoring THEW[73]	Average Heart rate detection	560ms	550ms	9.7 s only for additive operation as it is partial and additive homomorphic scheme		–	Intel-Xeon workstation W3565 work with 4 cores and 8 threads, RAM 24GB running 64-bit Ubuntu 15.04
[87]	–	LQTS, Average Heart Rate	635.42ms, 907.86 ms, 1254.71 ms, 1721.49 ms, 2241.55 ms with security parameter 200, 250, 300, 350, 400 respectively				–	Two Intel (R) Core(TM) i5-3470 CPU processors, frequency 3.20 GHz, RAM 8.00 GB, and 4:00 GB Virtual memory storage allocated, Operating system with single processor Ubuntu 12.04
[89]	i2b2 demo dataset from three different sites total of 32,301 rows	No Disease Prediction only querying of Data	13.77ms including interactive two party encryption protocol	0.9 ms	–	–	0.004ms Homomorphic aggregation	Intel Xenon E5645 processor with frequency 2.40GHz and RAM 4 GB
[90]	Sensor data of 45 patients using Polar H7 device and 500 patient dataset	Delayed Repolarization of Heart Syndrome	Performance time is 0.35 in 24 h monitoring data with 2079 cipher texts and 7.3 GB uploaded and 9.6 GB downloaded data including storage ratio of 53.6				–	Data stored in IBM Cloudant infrastructure homomorphic processing is done in Apache spark service in IBM Bluemix Platform and for event based handlers IBM OpenWhisk programming service
[94]	Simulation dataset based of statistical data by referring data given by Japan ministry of Health, Labour and Welfare	Query Generation	When no threading was in use then total Time is 236.72 s with FHE time is 195.82 s and with 28 maximum 28 threads total time was 61.74 s with FHE execution time at 11.33 s				–	Client Server equipped with Intel 8-cores i7-8770 CPU with RAM 16 GB, frequency 3.20 Ghz and cloud server with 28 virtual CPUs and Physical layer Memory of 256 Gb and 28 Intel Xeon model E52697-A v4 CPU with frequency 2.60 GHz, Ubuntu linux 18.04 OS used is n both setups
[95]	Simulated dataset of 5000 patients	Identification of exceptional survivor and Identification of drug exposures	1583.8 s with 256 bit security (exceptional survivor) and 56.8 s (drug exposure)	0.8 s for both exceptional survivor and drug exposure	2512.2 s (exceptional survivor) 5.3 s (drug exposure) for one patient	25,120 s (exceptional survivor) and 42.4 s (drug exposure) for one patient	–	–

**Table 6:** Comparison of homomorphic encryption in healthcare based upon computation and communication overhead

Paper	Base Approach	Type of HE	Computation overhead analysis	Communication overhead analysis	Overall analysis
[72]	Gentry [3]	Ideal Lattices	Requires an extra Recryption operation that requires 99.9 % of execution time	Nearly 4.8 GB of Storage Space is required for one hour patient encrypted ECG record. Communication cost increases due to storage overhead	In terms of computing and storage, it is inapplicable for a long-term monitoring system
[72]	BGV [43]	Leveled homomorphic using modulus switching (RLWE)	24-hour Average heart rate of patient can be calculated in around 70 ms	Storage of 24 hrs patient records requires around 28 MB. Communication overhead is less comparison to Gentry FHE	In terms of computing and storage BGV scheme is acceptable as it requires very less storage and there is no recryption operation
[74]	Stehle [52] Lopez [51]	NTRU and Leveled homomorphic using modulus switching (RLWE)	Computational Cost with Parameter set P1 ( to allow computation in logistic regression function) is 64 ms	Size of a single encrypted value using parameter P1 is 64 KB. Communication overhead is more	Although Communication overhead is more but still it is practical to implement this approach
[74]	Stehle [52] Lopez [51]	NTRU and Leveled homomorphic using modulus switching (RLWE)	Computational Cost with Parameter set P2 ( to allow computation of Taylor Series up to degree 7) is more as 5 s of time is required in ciphertext multiplication and Taylor computation up to degree 7 requires more than 30 s	Size of one ciphertext grows to 1 MB. Communicational overhead is huge	Both Computational and Communicational cost is high due to huge size of ciphertext and multiplication operation cost
[77]	BGV [43] HElib[55]	leveled homomorphic using modulus switching (RLWE)and HElib library	When data is delivered one minute at a time, the computational cost is smaller. With an FHE level of 7, the computation time for one chunk is 3.9 s. FHE level 18 computations are slower with whole 24 h data, i.e. 2093 chunks	The cost of communication lowers as the degree of FHE increases, as seen by the fact that 24 h data requires 4.1 MB of findings to be sent to the doctor, but 1 min data requires 1296.1 MB	Despite the fact that performance is not up to par, healthcare organizations may use these services because many cloud service providers only charge for incoming data, i.e. sending encrypted results data back to the doctor will cost, but sending encrypted data to the cloud will not, and communication costs will be reduced as the level of FHE is increased
[85]	[105]	NTRU	An optimized NTRU based HE results with less noise and high speed using GPU implementation (*GM*204 *Maxwell* *architecture*) results in a speedup of 6085x for ciphertext multiplication operation	Dividing the work among all four GPUs achieved a speedup of 3.946x, resulting into hiding almost all of the communication cost by splitting large problems into small ones	A huge speedup with less communication overhead is achieved. The results were compared with other RLWE and NTRU schemes
[86]	Paillier[15]	Partial homomorphic (Additive)	Average heart rate Computed using paillier Algorithm in which addition operation requires 0.11 ms and average heart rate for 24-hr data requires 9.7 s. While Encryption and Decryption operations were performed in 560 and 550 ms respectively	Ciphertext with 12,288 integers. Security parameter with 128-bit requires 3072-bit primes	Computation time of Paillier for Average heart rate detection is 3100 times faster then BGV but due to partial homomorphic property it is restricted only to addition operation. However, Encryption and Decryption time are slow in both the algorithms. Storage Requirement is 96 times more than AES algorithm
[86]	BGV[43]	Leveled homomorphic using modulus switching (RLWE)	For Average Heart Rate with 24 hr Monitor Interval, slots 2198 and level 31 Encryption and Decryption requires 1.8 and 0.85 s respectively with execution time of 502 min and LQTS detection with Slots 2093 and Level 18 requires 0.26 s for Encryption and 0.25 s for Decryption with execution time of 1165 min	For Average Heart Rate with 24 hr Monitor Level, slots 2198 and level 31. Size of ciphertext is 20.3 MB and for LQTS detection with same specification the size of ciphertext is 4.3 MB	Both LQTS and Average heart rate computation requires higher value of Level for longer ECG monitoring As a result it requires higher number of ciphertexts thereby requiring less network traffic for better communication
[87]	Dowlin [88]	Ring-Learning With Errors (RLWE)	In comparison to Kocabas [86] technique, less computation is required to detect LQTS due to efficient l-bit comparator, which requires just one homomorphic multiplication with l-Xor operations	The communication cost of computing average heart rate is reduced since only one ciphertext is provided to the receiver, as opposed to two ciphertexts containing the cumulative total and number of samples in Kocabas approach [86]	With security parameters ranging from 200 to 400, the implementation time for detecting long QT Syndrome has been significantly reduced. When computing average heart rate, the overhead of ciphertext is also decreased
[89]	El Gamal [11]	Discrete Logarithms	The computation load is linear since it employs the El Gamal partial homomorphic technique, which takes 13.77 ms to encrypt and 0.9 milliseconds to decrypt. With 0.004 ms, additional homomorphic aggregation is required for an aggregated Query	Communication overhead is 576 ms i.e the time for sending query and receiving encrypted results	Although the storage cost is higher, it is still feasible because the encrypted version is just 4x the size of the unencrypted version
[90]	BGV[43]	Leveled homomorphic using modulus switching (RLWE)	With 2079 slots/ciphertexts, the processing speed at Level 17 and 24 hr monitoring is 0.35, which is computed by dividing the time required to transfer data from the client to the backend by the time required to process the data at the backend. This demonstrates that the cost of computing is lower	The communication overhead for calculating Delayed Repolarization of Heart Syndrome diminishes as the level of granularity increases, requiring 1102 GB of data to be transferred for 1 mint monitoring and 9.6 GB for 24 hr monitoring	Healthcare organizations may use these services because many cloud service providers only charge for incoming data. For example, sending encrypted results data back to the doctor will cost, but sending encrypted data to the cloud will not, and communication costs will decrease as the granularity of FHE is increased
[94]	Smart and Vercauteren [32]	SIMD Style FHE	Without Threading with filtered database of 4911 total FHE execution time 195.82 sec and with Threading FHE execution time 11.33 sec	Without Threading communication time 43.18 sec and with threading communication time 50.17 sec	A very time efficient FHE query system. Communication cost is higher with or without threading but still practical
[95]	Fan and Vercauteren[46]	Lattice based leveled homomorphic (RLWE)	Computation time depends upon a threshold of survival duration. Computation time are longer compare to standard analysis	With increase in multiplicative depth. More communication cost is incurred	More computation time is required. In comparison to delays caused by a lack of learning from real-world events, computing time is insignificant. Patients and doctors will be more likely to engage in research if they know the data from their health records will never be decrypted, resulting in faster learning owing to improved statistical power

*KeyGen*$(1^\lambda )$: consider two polynomials $f_{1 \times 1},g_{1 \times 1} \leftarrow D_{\mathbb {Z}^n, \sigma _k}$ with condition *f* is invertible in ring $R_q$ and *f* $\equiv$ 1(*mod*2). Public key $p_k$ and secret key $s_k$ can be computed as:

$$\begin{aligned} p_k&={h_{1 \times 1}}\\&={g_{1 \times 1} \cdot f_{1 \times 1}^{-1} \in R_q} , and\\ {s_k}&={f_{1 \times 1} \in R_q} \end{aligned}$$

*Enc*$(p_k,m)$: $R_q$ is the message space and $\mu \in R_q$

$$C_{l \times 1}=\mu \cdot BDI(I_{l \times l}) + S_{l \times 1} \cdot h_{1 \times 1} + E_{l \times 1}$$, with $$S_{l \times 1}, E_{l \times 1} \leftarrow D_{R_q^{l \times 1},\sigma _c}$$

*Dec*$$(s_k, C)$$:

6 $$\begin{aligned} {{C_{l \times 1} \cdot f_{1 \times 1}}}&{={(\mu \cdot BDI(I_{l \times l})+ S_{l \times 1} \cdot h_{1 \times 1} + E_{l \times 1})\cdot f_{1 \times 1}}}\nonumber \\&= \mu \cdot BDI(I_{l \times l})\cdot f_{1 \times 1} + S_{l \times 1} \cdot h_{1 \times 1} \nonumber \\&\quad + E_{l \times 1}\cdot f_{1 \times 1}\nonumber \\&=\mu \cdot BDI(I_{l \times l})\cdot f_{1 \times 1} + S_{l \times 1} \cdot g_{1 \times 1} \nonumber \\&\quad + E_{l \times 1}\cdot f_{1 \times 1}\nonumber \\&{={\mu \cdot BDI(I_{l \times l})\cdot f_{1 \times 1}+err_{i \times 1}}} \end{aligned}$$

Decrypted ciphertext can be reformulated as $l \times 1$ polynomial vectors (Eq. 6) like $\mu f, 2\mu f,\cdot \cdot \cdot \cdot , 2^{l-1}\mu f$ along with some error vector. In [86] author uses the medical cyber-physical system to target remote health monitoring system for patients that sends ECG data from patient’s home to the cloud. Patient data is encrypted using Paillier [15] and BGV [43] scheme. Paillier scheme is used to detect Average heart rate as it is only additive homomorphic, but BGV is used to detect LTQS (Long QT syndrome). THEW [73] (which is 24 − hour anonymized ECG recordings of real patients), with a sampling rate of 1000 Hz. 128-bit security for recommending encrypted data was used. LQTS implementation was compared based on bit-length of *toc* values in the dataset. Encryption and Decryption time using both Paillier and BGV is almost the same, but when it comes to evaluation Paillier becomes 3100 $\times$ faster. However, Paillier is a partial homomorphic scheme, i.e., only additive homomorphic. In [87] author proposed a four-layer architecture of the mobile health network, including wearable area, pre-processing area, cloud server area, and physical diagnosis area, followed by safe average heart rate calculation, long QT syndrome, and chi-square detection. Dowlin’s protocol is used to determine the average heart rate and long QT syndrome and uses chi-square tests to check the relation between varicose veins and overweight patients. Dowlin FHE scheme [88] consists of:

*n* *as security parameter*=$$2^k$$ where *k* $$\in $$, *q* *is large prime number with* $$q mod n \equiv 1 $$, *t* *is small plain text modulus where* $$1<t<q $$, $$\chi _{key}$$ and $$\chi _{err}$$ are randomly chosen from $$\overline{\psi \beta }$$,*params=(n,q,t,* $$\chi _{key}$$ *and* $$\chi _{err}$$)

*KeyGen(params)* keygeneration sample polynomial *f*’*g* $\leftarrow$ $\chi _{key}$

$f=[1+tf$’$]_q$ *if f is inverted modulo it computes*$f^{-1}$ of *f* in $R_q$, otherwise it chooses new *f*’.

Generate vectors *e* and *s* $\in$ $R^l$ randomly, in which every component selected from $\chi _{err}$.

$$\gamma $$=$$[Powersof2(f)+e+hs]_q$$

$$h=[tgf^{-1}]_q$$ public key $$p_k=h$$, secret key$$ s_k=f$$ and evaluation key $$ev_k$$=$$\gamma $$

*Enc*$$(p_k,m \in R_t)$$:

$$c=[\lfloor q/t \rfloor m +e_2 +hs_2]_q$$

*Dec*$$(s_k,c)$$:

$$m=[\lfloor t/q \cdot [fc]_q \rceil ]_q$$.

In [89], author demonstrated efficient use of homomorphic encryption for computing aggregate queries on ciphertext and also provides confidentiality of aggregate-level data from *i*2*b*2 data model. A privacy-preserving data sharing approach based upon El Gamal cryptosystem [11] which is additive homomorphic. It includes three phases, first is *Initialization Phase* to generate public and private keys for EL Gamal Cryptosystem followed by *ETL Phase* for encrypting i2b2 aggregate-level. After that, there is a *Querying Phase*. A query can be generated after, the encrypted data are stored on the storage server. With every query fresh temporary pair of keys are generated. After receiving the query, the server fetches the records encrypted under the public key and uses the crypto engine for homomorphically aggregating them. It also includes an interactive two-party encryption protocol with a proxy server for switching encryption of results. In [90], a SafeBioMetrics system architecture was proposed that enables secure cloud storage and processing of medical data using BGV [43] fully homomorphic encryption scheme. The Polar H7 device was applied as an experimental population sample to 45 individuals, and additionally dataset of 500 patients was used to detect DRHS *(Delayed Polarization of Heart Syndrome)* medical condition. Another i2b2 data privacy and security approach were proposed [91]. Privacy solution is integrated on top of the i2b2 framework using homomorphic encryption and differential privacy. Proposed solution was based upon Fan and Vercauteren cryptosystem [46], which is *lattice based leveled homomorphic scheme with RLWE (Ring Learning with errors)* enabling privacy based queries on the genomic data. The implementation of genomic cohorts in a real-world scenario took place at a hospital. Performance was evaluated with 707 MB of data that includes nearly 18.5 crore genotypes with each genotype of 4 bytes. The BGN [23] homomorphic cryptosystem has been used [92] as a cloud-based online medical prediagnosis approach that is both efficient and private. It enables the system’s online prediagnosis function, which may instantly be executed on the ciphertext. In [93], a medical encryption algorithm was proposed based upon elliptic curve cryptography combined with homomorphic encryption. Traditional ECC was expensive due to inversion operation, which is ignored in its improved version. The effectiveness of the algorithm was verified upon two medical images which show better keyspace and higher key sensitivity.

In [94], a privacy-preserving query model system was proposed, and put into use in a real-world medical side effect inquiry system. To reduce the size of database, a filtering technique was applied before query model deployment. Multi-threading was used for fast searching. The effectiveness of the approach was verified 10,000 times with a random query, using a database including 40,000 records of simulation data. Nearly about 99% of the queries are completed within 60 s. In [95], homomorphic encryption by Fan and Vercauteren [46] was implemented using R language on a simulated dataset of 5000 cancer patients with a security level of 128 bit. Identification of exceptional survivors is made with the identification of total drug exposure.

In [96] study adds security mechanisms into the architecture of a patient-oriented edge-cloud-based healthcare system. For secure processing and filtering of insensitive data processing in the edge layer, an edge-level additive homomorphic encryption is presented. In [97], using contrast-enhancement RDH and homomorphic encryption, a safe and high-visual-quality framework for medical pictures was created. The proposed algorithm was homomorphic encryption based upon a chaotic map. It was a partial homomorphic scheme with the additive homomorphic property. The correlation between encrypted and marked photos is found to be lower, as well as the unpredictability in encrypted images is fairly high. It implies that the proposed encryption approach can enhance image security by safeguarding image content. In [98], proposed a computational model and developed an algorithm for contract tracing with COVID-19 pandemic. The proposed algorithm was simulated using ElGamal [11] encryption algorithm. Simulation was done using a synthetic spatiotemporal geolocation dataset. It was generated with timestamped locations. It consists of all location-oriented information for more than 24 h. It also includes ten COVID-19 positive patients and one querying person. It covers region $400\,Km^2$ within 24 hrs duration. Threshold distance of 2 m and Threshold time duration of 15 min with a threshold time difference of 12 h. According to simulation findings, the proposed approach effectively ensures security while also meeting the computing requirements for contact tracing. MHE allows for flexible, secure processing by seamlessly switching between HE-encrypted local computation and interactive protocols (SMC). It can be used to determine the most efficient strategy for each phase in a workflow, making use of one technique’s features to avoid the bottlenecks of the other. A self-serviced medical diagnosis system based upon homomorphic encryption (HE) and privacy-preserving access control was described [99], which may protect patient medical health data while keeping hospital diagnosis mode secret. Following a thorough security investigation, it was discovered that the scheme could withstand a variety of known security risks. To [100] handle medical data privacy problems in smart health, the author tackled two major issues in the adversarial model. First, it created Derepo to decentralize access control and encryption of data without losing computability. Static structures and dynamic processes are formalized as part of the double-layered architecture. Internal and external adversaries pose privacy problems. Thus the FHE method is employed to maintain secrecy and handle them. The author classifies data-sharing models into three groups and examines the major technical difficulties faced. Medical sites (i.e., data providers) that are willing to share data must pool their individual-level patient data into a single repository under the *centralized* approach. Unlike the centralized data-sharing approach, the *decentralized* model does not necessitate the physical transfer of patient-level data from medical locations’ IT infrastructure. For calculating average heart rate, computing minimum and maximum heart rates, and detecting LQTS, the author [101] employed the BGV [43] scheme and its practical implementation HElib [55]. Because the BGV system employs leveled FHE, it avoids the need for a costly recryption procedure. For handline noise, leveled FHE employs a better technique known as modulus switching. Numerous ways that are primarily concerned with the privacy of healthcare data using Homomorphic Encryption are compared in (Table 5 and Table 6), and it was determined that, despite performance concerns owing to large computation, homomorphic encryption is feasible and important for preserving patient data privacy. In [102], authors introduced a FAMHE scheme, which is a federated analytics system based on multiparity homomorphic encryption that integrates the strength of HE for executing computations on encrypted data without requiring communication between the parties. Safe computing and learning involve combining federated learning approaches with cryptographic frameworks. The technique was demonstrated to provide stringent privacy protection, but the accuracy of the approach was sacrificed in favor of performance. In [103], authors proposed two schemes CTHE *( Carmichael’s Theorem-based Homomorphic Encryption)* and MEHE *(Modified Enhanced Homomorphic Encryption )*. The hardness of integer factorization, the discrete logarithmic approach, and quadratic residuosity were employed in both techniques. It also makes use of modulus switching to decrease noise. Although the overall number of computations in MEHE was not decreased, the approach is still superior since it decreases noise without impacting security. In [104], authors presented a privacy-preserving framework CFHET-PPF. It combines three FHE approaches resulting in the prevention of false data injection. It is presented as a way to make it easier for fog nodes to categorize shared data based on illness risks, which is necessary for health data analysis. Because it prevents irrelevant computing during the transmission of sensitive data disseminated in the fog computing environment, it reduces encrypting time.

## Conclusion

Privacy of data is essential than ever before in this internet world. Outsourcing applications are in high demand due to the cloud computing revolution. Clients use the service by uploading their data to the cloud, which is then evaluated to get a result. It benefits the consumer and exposes critical data to third-party service providers. Although data are encrypted, the difficulty with encrypted data is that it must be decrypted before being used. As a result of decrypting, it becomes vulnerable to all the things that were trying to secure it. Homomorphic encryption, which processes data while maintaining privacy and security, maybe the greatest solution for this problem in the future. The concept of HE has been around for more than three decades, but rapid development in the subject of fully homomorphic encryption advanced the field swiftly from Gentry’s innovative research to today’s efficient implementations. Digitizing a patient’s medical records is anticipated to increase the quality and effectiveness of care while also lowering expenses. However, because electronic health records (EHRs) include a vast quantity of sensitive data available not just to doctors but also to a range of other sources such as insurance companies and staff members. EHR administration is essential for data security issues. As a result, we surveyed and compared the HE and FHE schemes in this paper. Initially going with the fundamentals of HE, the concepts of Partial Homomorphic Encryption (PHE) and Somewhat Homomorphic Encryption (SHE) were examined, which are prime aspects in achieving Fully Homomorphic Encryption, followed by FHE. FHE approaches were divided into four distinct groups, and significant approaches in each group were compared. Finally, in the domains of healthcare and bioinformatics, HE techniques were retrieved and compared for securely identifying LQTC, cancer, average heart rate, cardiovascular problems, and a secure query generating system in healthcare. Overall, homomorphic encryption will be a boon to the healthcare business as the performance and utility of FHE improve.
